# Neoadjuvant Immunotherapy Combined with Chemotherapy for Local Advanced Non-Small-Cell Lung Cancer in a Patient with a History of Breast Cancer: A Case Report

**DOI:** 10.3390/curroncol29090487

**Published:** 2022-08-29

**Authors:** Rui-Xia Yang, Yue Hei, Wen-Ting Zhu, Qian-Rong Wang, Hong-Mei Zhang, Yan Chen

**Affiliations:** Department of Oncology, Xijing Hospital of Air Force Military Medical University, Xi’an 710032, China

**Keywords:** Neoadjuvant immunotherapy, non-small-cell lung cancer, pathologic complete response

## Abstract

Durvalumab consolidation therapy is the standard treatment after concurrent chemoradiotherapy for patients with surgically unresectable stage IIIA (N2) non-small-cell lung cancer (NSCLC). Neoadjuvant therapy followed by surgery could reduce locoregional and distant recurrence and improve the survival rate for surgically resectable NSCLC. However, the value of neoadjuvant therapy in locally advanced potentially resectable NSCLC remains controversial. Herein, we report a locally advanced potentially resectable NSCLC case with a history of breast cancer who achieved a pathologic complete response (pCR) after preoperative treatment with pembrolizumab and chemotherapy. A 50-year-old woman developed squamous cell carcinoma (SCC) (left lower lobe of the lung, stage IIIA-N2) after two years of chemotherapy and anti-HER2 therapy following a diagnosis of HER2-overexpressing breast cancer. Surgical resection was attempted despite an MDT classification as unamenable to curative surgical resection. After two cycles of neoadjuvant chemotherapy combined with anti-PD1 immunotherapy, the tumor significantly shrank, then the patient underwent a left lower lobectomy. Complete resection with negative margins (R0 resection) was achieved in the patient. The patient experienced grade 1–2 adverse effects and no grade 3 or worse adverse effects occurred. Cardiotoxicity did not occur in the patient despite prior anti-HER2 treatment for breast cancer. Our case study contributes to the existing evidence on the feasibility, efficacy, and safety of neoadjuvant immunotherapy combined with chemotherapy in locally advanced unresectable NSCLC. Furthermore, future studies are needed to determine which patients can benefit from immunoadjuvant therapy and the duration and course of preoperative and postoperative immunotherapy.

## 1. Introduction

Recent research indicates that lung cancer remains the leading cause of cancer-related mortality worldwide [1], with approximately 2.2 million newly diagnosed cases and 1.8 million deaths reported annually [2]. Non-small-cell lung cancer (NSCLC) accounts for 85% of all lung cancers and approximately 25–40% of NSCLC patients are locally advanced (stage III) at diagnosis [3]. Stage III NSCLC is a highly heterogeneous group of diseases with different presentations and subgroups (stage IIIA/B/C) depending on the tumor and nodal status, which can be categorized into resectable, potentially resectable, and unresectable diseases. Approximately 30% of locally advanced diseases are unresectable, with a 5-year survival rate of 15–20% [4]. However, a portion of stage III cancers fall into the potentially resectable category. Surgical resection in combination with neoadjuvant therapy can improve patient survival rates. The role of neoadjuvant immunotherapy in patients with resectable stage I–IIIA cancer has been demonstrated in multiple clinical studies but remains controversial in potentially resectable III NSCLC. Herein, we describe a patient with potentially resectable lung squamous cell carcinoma, who had a past history of radical breast cancer surgery and underwent chemotherapy and anti-HER2 treatment. A pathologic complete response was achieved after neoadjuvant pembrolizumab combined with chemotherapy, suggesting that neoadjuvant immunotherapy might be beneficial in selected potentially resectable stage III NSCLC patients.

## 2. Case Presentation

**Figure 1:**
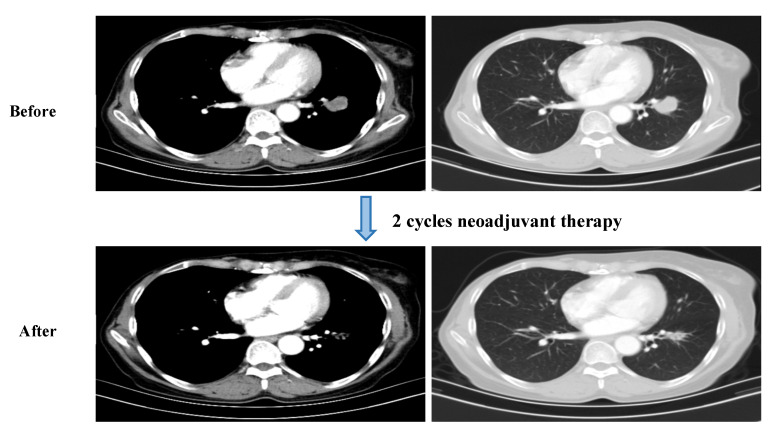
Computed tomography scan of the patient before and after neoadjuvant therapy showed the tumor significantly shrank.

In July 2021, a 50-year-old Chinese woman presented to the clinic due to a productive cough and hemoptysis for more than a month. She had moderate mixed ventilatory dysfunction despite not having a smoking history. Enhanced chest CT on 25 July 2021 revealed the presence of a 2.1 cm × 2.8 cm malignant mass in the left lower lobe and mediastinal and multiple left hilar lymph node metastases (1.7 cm) with obstructive pneumonia (see Figure 1). Notably, she had a history of breast cancer and received a modified radical mastectomy on 8 March 2019. The pathology revealed infiltrating ductal carcinoma of the right breast at stage IIA with a subtype of hormone-receptor negative and HER2 overexpression. The patient also received postoperative adjuvant therapy (sequential doxorubicin/cyclophosphamide followed by paclitaxel and trastuzumab (AC-TH)) and trastuzumab maintenance treatment until April 2020. No adjuvant radiotherapy was performed postoperatively. Routine examination showed no recurrence or metastasis. Given her previous history of breast cancer, it was necessary to clarify whether the patient’s left lung lesion was secondary cancer. Therefore, on 4 August 2021, we scheduled a bronchoscopic lung biopsy for the patient and the biopsy pathology revealed carcinoma tissue that presented a solid sheet arrangement with a squamous cell phenotype. The immunohistochemistry showed the following staining patterns: CK7 (−), P40 (+), TTF-1 (−), GATA-3 (−), GCDFP-15 (−), ER (−), PR (−), HER-2 (0), KI-67 (70%); PD-L1 (SP263; TPS = 30%). EGFR, ALK, ROS1, RET, BRAF, HER-2, MET, KARS, and PIK3CA genomic aberrations were not identified by next-generation sequencing (NGS) in the primary lesion. Based on the pathological examination and imaging tests, the patient was diagnosed with lung SCC (left, stage IIIA-N2) without the driver gene mutation. We held a multidisciplinary discussion for the patient in August 2021. MDT suggested that the cancer was a potentially resectable stage III NSCLC that might respond to neoadjuvant therapy. Given this, we decided to give the patient two cycles of preoperative neoadjuvant immunotherapy combined with chemotherapy (pembrolizumab (200 mg) and carboplatin (AUC5)/nab-paclitaxel (125 mg/m^2^) for 3 weeks). The initial cough and hemoptysis were completely resolved.

**Figure 2:**
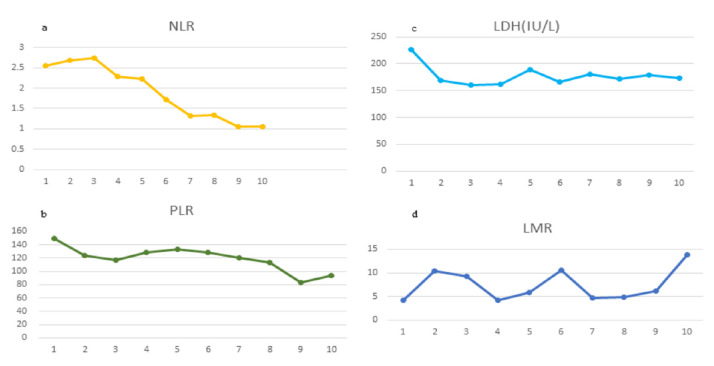
(**a**) indicates the decreasing trend of the NLR; (**b**) indicates a decrease in the PLR; (**c**) indicates a higher level of serum LDH at the time of initial diagnosis, which decreased after treatment; (**d**) indicates a significant fluctuation in the LMR.

A partial response (PR) was determined based on a CT scan evaluation following the RECIST version 1.1 criteria, which showed a reduction in the size of the left lower lobe lesion and the previously documented mediastinal lymph node after the second cycle of therapy (see Figure 1). The patient was monitored until CEA gradually decreased to a normal level. These levels changed from 8.32 ng/mL of CEA on 4 August 2021 to 5.17 ng/mL on 9 September 2021 and 2.31 ng/mL on 10 December 2021 (reference value 0–5 ng/mL). At the same time, we collected clinical data from patients administered pembrolizumab and analyzed the data to determine the changes in the routine laboratory data (see Figure 2), such as the neutrophil–lymphocyte ratio (NLR), lymphocyte–monocyte ratio (LMR), and platelet–lymphocyte ratio (PLR). We collected pretreatment NLR (NLR-0), LMR (LMR-0) and PLR (PLR-0), NLR-2, LMR-2, and PLR-2 (2 weeks after the start of pembrolizumab), NLR-4, LMR-4, and PLR-4 (4 weeks after the start of pembrolizumab). The NLR (Figure 2a) and the PLR (Figure 2b) exhibited a gradually decreasing trend and the serum lactate dehydrogenase (LDH) decreased from 226 IU/L (>185 IU/L) at the initial diagnosis to 174 IU/L (<185 IU/L) (Figure 2c). However, the LMR fluctuated greatly during treatment without a significant trend (Figure 2d).

**Figure 3:**
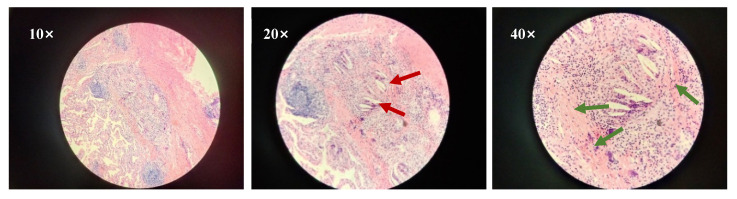
Hematoxylin–eosin staining of pathological results of postoperative specimens.

**Figure 4:**
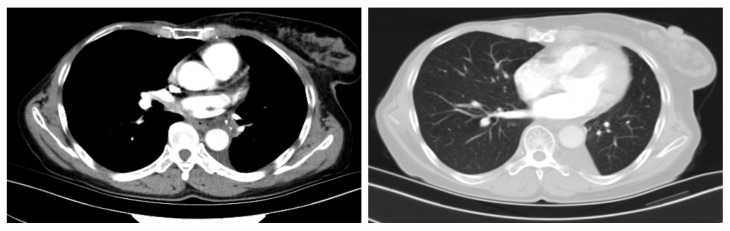
A follow-up CT scan revealed postoperative changes with no evidence of disease recurrence.

**Figure 5:**
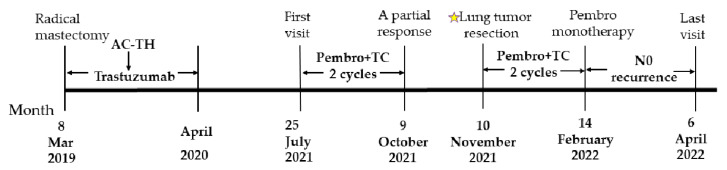
A patient treatment timeline.

The combination therapy was well tolerated and the patient experienced grade 2 myelosuppression and grade 1 thyroid dysfunction, which were both controlled via symptomatic interventions. Adverse events were graded based on the common terminology criteria for adverse events (CTCAE-version 5.0). Considering the potential cardiotoxicity due to adjuvant chemotherapy and anti-HER2 therapy following radical breast cancer surgery, we focused on evaluating the patient’s electrocardiography (ECG), myocardial enzyme spectrum, and left ventricular ejection fraction (LVEF) and other related indicators. No cardiac adverse effects have been observed in the patient to date. The patient underwent a thoracoscopic left lower lobectomy with lymph node dissection on 10 November 2021; R0 resection was achieved with negative margins. No residual cancer cells were observed in the resected pulmonary tissue and lymph nodes during the postoperative pathological examination. Instead, chronic inflammatory tissue hyperplasia, multinucleated giant cells, a cholesterol crystal, and focal calcification were observed (Figure 3). The postoperative pathology of the patient indicated that she had a pathological complete response (pCR). The patient accepted two cycles of adjuvant chemoimmunotherapy with carboplatin/nab-paclitaxel and pembrolizumab, followed by pembrolizumab maintenance therapy. She is currently in good condition and has not exhibited any grade 3 or higher adverse reactions. A follow-up CT scan in April 2022 revealed postoperative changes, with no evidence of disease recurrence (see Figure 4). There is a treatment timeline of the patient (see Figure 5).

## 3. Discussion

In recent years, despite the improvement in patient survival, the incidence of multiple primary malignant tumors (MPMNs) has gradually increased to 0.73–11.7% [5]. There is a heightened risk of developing second primary cancers in patients with primary malignant tumors compared with the general population [6]. In addition, MPMNs have increased malignant behavior and an overall worse prognosis [7]. In our case, the patient received breast surgery in 2019 and had her second primary tumor develop 2.5 years later. According to the literature, postoperative radiotherapy for early breast cancer probably increases the risk of second primary tumor development and the risk continues to increase until 15 years after surgery, particularly second primary lung cancer for whole breast radiotherapy [8,9,10]. Therefore, patients with a previous history of breast cancer should be screened for the presence of second primary tumors.

In this case, we describe a patient with NSCLC (left, stage IIIA-N2), with a 5-year survival rate of 19–36%, as recorded. Stage IIIA/B-N2 is a significantly heterogeneous group of patients and accounts for one-third of NSCLC at diagnosis [11]; thus, according to a consensus reached in terms of combined modality by the Asian Thoracic Oncology Group, a clinical assessment with MDTs is necessary [12]. Approximately 30% of patients with locally advanced (stage III) disease are unresectable and the considerations for potentially operable NSCLC include tumor size, lymph node involvement, and the presence of distant metastasis [12]. The current standard for unresectable stage III NSCLC is the PACIFIC modality. The 5-year survival rate of patients with stage III NSCLC has increased from 9–24% to 12–41% [13]. On the other hand, induction or neoadjuvant immunotherapy (with or without chemotherapy), followed by re-evaluating the possibility of surgery, has also been suggested. In our case, the patient underwent neoadjuvant therapy followed by radical surgical treatment according to the MDT. Several points of the decision need to be addressed regarding preoperative therapy options for this patient.

As far as we know, whether or not to use systemic chemotherapy alone (or combined with radiotherapy) is still a tough choice for patients at stage IIIA/N2. Specifically, in a prospective randomized study of 232 patients with NSCLC (stage IIIA/N2), the Swiss Group for Clinical Cancer Research demonstrated the similarity between pCR and lymph node descent rates in the chemotherapy-alone group and the combined group [14]. In the present study, we included the following concerns: (i) Preoperative treatment is recommended for patients with stage N2 disease or higher. (ii) Sequential chemoradiation therapy does not provide an additional benefit over chemotherapy alone. (iii) The patient is young and has a strong desire for surgery.

Second, we administered neoadjuvant chemoimmunotherapy rather than chemotherapy. Preoperative chemotherapy, as reported, can shrink tumors, improve complete resection rates, decrease postoperative recurrence rates, and provide better survival outcomes than surgery alone [15,16]. However, the benefit of chemotherapy is limited and there is an urgent need for well-tolerated and effective neoadjuvant therapies. A recent meta-analysis based on perioperative clinical data demonstrated that neoadjuvant immunotherapy was feasible, safe and had a long-lasting effect. Some studies demonstrated an obvious and permanent increase in antitumor T cells in peripheral blood (even after the primary tumor was removed) using neoadjuvant immunotherapy [17]. In addition, some trials indicated the presence of a synergetic effect of neoadjuvant immunotherapy and chemotherapy [18]. The CheckMate 816 trial (ClinicalTrials.gov identifier NCT02998528), in which 358 patients with stage IB0-IIIA NSCLC were randomized to receive either three cycles of neoadjuvant chemotherapy or three cycles of chemotherapy with nivolumab, reported a major pathological response (MPR) rate of 36.9% and a pCR rate of 24% [19]. This pattern significantly improved the outcomes in IIIA NSCLC patients with multi-station N2 lymph node metastases [20]. The trial presented promising implications for the safety of this regimen.

Furthermore, the efficacy of immune-checkpoint inhibitors (ICIs) varies greatly among different tumor types and individuals. Therefore, identifying reliable biomarkers as predictors of clinical response and resistance to ICIs has become urgent. PD-L1 expression [21] by tumor cells and tumor mutation burden (TMB) [22] are included in the National Comprehensive Cancer Network (NCCN) guidelines. Furthermore, some scholars included high PD-L1 expression, positive KRAS + TP53 mutations [23], negative EGFR/ALK alterations, high infiltration of CD8-positive cells, and observation of M1 macrophages—each being a favorable characteristic for immunotherapy [24]. Similarly, our case presents a squamous cell carcinoma phenotype, high PD-L1 expression (TPS = 30%), and absence of EGFR/ALK driver alterations. It has been clarified that the baseline and variations in NLR, LDH [25,26], LMR [27], and PLR [28] are predictors of immunotherapy for determining whether there is early hyperprogression of tumors. Several studies have shown that a low NLR and PLR and a high LMR at baseline are favorable for longer progression-free survival (PFS). Others have found that CEA can predict efficacy. Therefore, in addition to imaging studies, we also monitored the patient’s NLR, PLR, LDH, and CEA levels. The above indicators suggest that patients respond to immunotherapy. However, the lymphomonocytic ratio LMR fluctuated greatly during the monitoring period, with no clear trend.

The time cost and safety of neoadjuvant therapy should be handled carefully. Generally, a complete response evaluation should be performed after two cycles of neoadjuvant therapy. For patients with a clearly resectable tumor, the delay of a potentially curable resection may negatively affect the survival rate, especially when the patient is a nonresponder. For such patients, oncologists should conduct assessments in time and operate as early as possible. Regarding safety, the patient we present has a history of HER2-overexpressing breast cancer and underwent adjuvant chemotherapy with anthracyclines, paclitaxel, and anti-HER2-targeted therapy. As these drugs have cumulative cardiotoxicity, it is important to evaluate and protect cardiac function during treatment.

## 4. Conclusions

This case has some limitations, with the follow-up time from surgery being too short and there are similar cases on neoadjuvant immunochemotherapy. We highlighted that patients with a previous history of breast cancer should be screened for the presence of second primary tumors and the clinical decisions of newly diagnosed unresectable driver-gene-negative NSCLC in stage III requires MDTs. For potentially resectable stage III NSCLC, immune neoadjuvant therapy holds curative promise. However, several avenues remain to be explored, including determining, which patients can benefit from this treatment, the timing and number of cycles of neoadjuvant therapy, whether postoperative adjuvant therapy requires intervention, and the duration of such an intervention.

## Data Availability

The original contributions presented in the study are included in the article. Further inquiries can be directed to the corresponding authors.
